# Oncological outcome and recurrence pattern analysis after involved-field irradiation in combination with rituximab for early-stage nodal and extranodal follicular lymphoma

**DOI:** 10.1007/s00066-020-01624-w

**Published:** 2020-05-06

**Authors:** Laila König, Klaus Herfarth, Juliane Hörner-Rieber, Sascha Dietrich, Thomas Wiegel, Jürgen Debus, Andreas Viardot

**Affiliations:** 1 Department of Radiation Oncology, University Hospital Heidelberg, Im Neuenheimer Feld 400, 69120 Heidelberg, Germany; 2 Heidelberg Institute of Radiation Oncology (HIRO), Heidelberg, Germany; 3 National Center for Tumor diseases (NCT), Heidelberg, Germany; 4 Department of Internal Medicine V, University Hospital Heidelberg, Heidelberg, Germany; 5 Department of Radiation Oncology, University Hospital Ulm, Ulm, Germany; 6 Department of Internal Medicine III, University Hospital Ulm, Ulm, Germany

**Keywords:** Radioimmunotherapy, Immunotherapy, Indolent lymphoma, CD20 antibody, Grade 3A follicular lymphoma

## Abstract

**Purpose:**

Combined radioimmunotherapy (RIT) in follicular lymphomas (FL) has shown promising treatment efficacy in the Mabthera® and Involved field Radiation (MIR) study. Aim of this study was to analyze treatment efficacy and recurrence patterns after RIT in early-stage nodal and extranodal FL.

**Methods:**

We reviewed 107 patients who were treated with combined RIT in two centers. Treatment consisted of 4 × rituximab followed by RIT with 4 × rituximab and involved field (IF) radiotherapy with 30/40 Gy. Median follow-up period was 71 months. In contrast to the MIR study, extranodal involvement and grade 3A histology were included in the analysis.

**Results:**

Extranodal involvement and grade 3A histology were present in 21.8% and 13.1%, respectively. Overall response rate (ORR) after 4 × rituximab, after completion of RIT, and after 6 months was 78.1%, 98.8%, and 98.8%, respectively, with increasing rates of complete remissions (CR). Predictive factors associated with superior PFS were tumor size, completely excised lymphomas, and response to first 4 × rituximab. 5‑year PFS rate was 87.3%, with mostly outfield recurrences (94.1%). Second-line treatment was effective, with 53.3% CR and 46.7% partial remissions (PR). 5‑year OS was 98.1%. RIT was tolerated well, with mainly grade 1–2 acute side effects.

**Conclusion:**

The real-world efficacy of RIT is comparable with the results of the MIR study. Additionally, this analysis shows that extranodal involvement and grade 3A histology are not associated with inferior PFS.

## Introduction

For patients with early-stage (Ann Arbor stage I–II) follicular lymphoma (FL), radiation therapy (RT) alone has been the gold standard. Long-lasting remissions and the potential chance of cure were the main arguments supporting this effective treatment. However, treatment has been discussed for many years [1]. Under the assumption that FL mainly spreads through the lymph nodes, patients were treated by large-field irradiation techniques to also encompass microscopic disease spread. Ten-year progression-free survival (PFS) rates in different studies of the pre-rituximab era, using involved field (IF), extended field (EF), and total lymphatic irradiation (TLI), ranged from 38 to 72% [2–8]. However, extensive radiation protocols are associated with significant toxicities, e.g., grade 3 and 4 adverse events concerning the hematopoietic system in 22% of patients [7]. Therefore, the National Comprehensive Cancer Network (NCCN) guidelines recommend radiation treatment of the pathologically involved regions only (involved-site radiation therapy [IS-RT]) without prophylactic treatment of additional lymph node areas. To further eliminate the risk of distant failure, several studies combined radiation therapy with systemic chemotherapy [9–12]. With development of the monoclonal chimeric anti-CD20 antibody rituximab, treatment of FL has been markedly improved. A recently published randomized trial showed a superior PFS with IF-RT and combined immunotherapy with R‑CVP compared to IF-RT only, showing that additional rituximab-comprising systemic therapy reduces out-of-field relapses and therefore might be an important component [13]. Besides, rituximab enhances radiosensitivity in vitro and may thus be an ideal combination partner to improve the efficacy of radiotherapy [14]. The hypothesis that rituximab in combination with IF radiotherapy might prevent out-of-field relapses in early-stage nodal FL was investigated in the prospective multicenter phase II MIR study [15, 16]. The combination of localized standard-dose radiotherapy and rituximab showed a high efficacy, with low recurrence rates and preserved quality of life [15].

This retrospective study evaluates the effect and toxicity of radioimmunotherapy (RIT) treatment analogue to the MIR study under real-world conditions and also conducts a detailed recurrence pattern analysis. In contrast to the MIR study, this cohort not only includes patients with nodal but also those with extranodal disease and histological grade 3a (WHO grading) FL.

## Materials and methods

### Patients and tumor characteristics

Patient records and follow-up (FU) imaging of 107 patients who had been treated with combined RIT according to the MIR concept were reviewed in two centers (University Hospital Heidelberg and University Hospital Ulm). Median age was 56 years (range: 23–82 years). All lymphomas had been histologically confirmed and staged with CT and bone marrow biopsy. Except for one patient, only patients with Ann Arbor stages I–II were treated. One patient had low-level bone marrow infiltration but with only isolated extranodal manifestation (intraspinal). Nineteen of the analyzed patients had been treated within the MIR study and are now analyzed with a longer follow-up.

In 25.2% (27/107) of the patients, the lymphoma had been excised completely, so that no macroscopic disease was seen at the time of treatment. The response in the remaining 80 patients was evaluated separately. Further details of patient, tumor, and treatment characteristics are shown in Table 1.

**Table 1:** Patients, tumor, and treatment characteristics

		Number of patients/lesions
*Total number*		107
*Age (years)*		
Median (range)		56 (23–82)
*Gender*		
Male		52 (48.6%)
Female		55 (51.4%)
*Histology Grading*		
Grade 1		50 (46.7%)
Grade 2		42 (39.3%)
Grade 3A		14 (13.1%)
n.a.		1 (0.9%)
*Stage (Ann Arbor)*		
I		66 (61.7%)
II		42 (39.3%)
III		0
IV		1 (0.9%)
*N/E manifestation*		
N		85 (79.4%)
E		22 (20.6%)
*Tumor site*		
N	Nodal	85 (79.4%)
E	Total	22 (20.6%)
	Salivary glands	13 (59.1%)
	Bone	1 (4.5%)
	Others	8 (36.4%)
*Initial size*		
≤7 cm		89 (83.2%)
>7 cm		18 (16.8%)
*Biopsy or excision*		
Biopsy		80 (74.8%)
Excision		27 (25.2%)
*Pattern of involvement*		
Singular		60 (56.1%)
Multiple		47 (43.9%)
*Full dose (8 cycles) rituximab applied*		
Yes		102 (95.3%)
No		5 (4.7%)
*RT technique*		
3D-conformal		76 (71.0%)
IMRT		31 (29.0%)
*RT dose*		
30 Gy in 15 fractions		50 (47.7%)
40 Gy in 20 fractions		57 (52.3%)

*N* nodal, *E* extranodal, *RT *radiotherapy, *IMRT* intensity modulated radiotherapy, *n.a.* not applicable

### Treatment and follow-up

Treatment was applied according to the MIR study [15, 16]. It consisted of four once per week administrations of rituximab (375 mg/m^2^) upfront. In week 7, patients received a restaging and radiation planning CT of the involved region. Four further weekly administrations of rituximab were given in weeks 9–12 during the radiation treatment period. Radiotherapy of the involved lymph node regions was initiated in week 9 and applied in 2 Gy single doses (five times per week) up to a total dose of 30 Gy. In case of remaining lymphoma after initial rituximab therapy in week 7, the residual region was boosted with an additional 10 Gy (5 × 2 Gy) in week 12 to a total dose of 40 Gy.

Patients were followed up regularly with clinical examinations and CT or MR imaging. Response evaluation was performed using the response criteria for lymphoma [17, 18] classified into complete remission (CR), partial remission (PR), stable disease (SD), and progressive disease (PD). Relapses were classified as infield or outfield, if the new manifestation was detected inside or outside of the radiation field, respectively. Toxicity was classified according to the Common Terminology Criteria for Adverse Events v4.03 (CTCAE) 8–12 weeks after RIT (acute toxicity) or 3–6 months after RIT (late toxicity).

### Statistical analysis and ethics

We reviewed patient records, planning documents, and imaging scans to assess response, current status of the disease, and following therapies. The median follow-up was calculated using the reverse Kaplan–Meier method [19]. Response was assessed at different timepoints: 1) after application of four cycles rituximab and before the start of radiotherapy (week 7); 2) at first follow-up (FU) 8–12 weeks after completion of RIT; and 3) after 6 months. Overall survival (OS) was calculated in months from the beginning of therapy until the last date of follow-up or death. Progression-free survival (PFS) was calculated in months from the beginning of therapy until the diagnosis of recurrent disease or death. The survival rates were displayed using the Kaplan–Meier method. Survival curves and response rates were compared between groups in univariate and multivariate analysis applying the log-rank test or cox regression analysis. All statistical analyses were performed using the software SPSS 24.0 (IBM Corporation, Armonk, NY, USA). The analysis was approved by the local ethics committee (S-106/2019).

## Results

### Patients and treatment

**Fig. 1:**
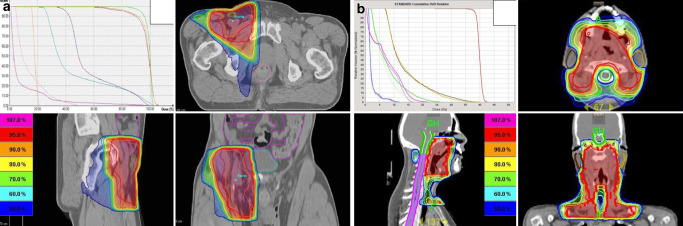
Representative RT plans of two patients treated with 40 Gy IF-RT. **a** Patient treated with 3D-conformal RT due to FL stage I (grade 2) with right-sided inguinal involvement. The residual lymph node can be seen as gross tumor volume in the transverse and sagittal screenshots. **b** Patient treated with helical IMRT due to FL stage II (grade 3A), with involvement of the nasopharynx and cervical lymph nodes. Due to the more extensive involvement and the necessity of sparing close organs at risk like the parotid glands, the patient was treated with IMRT

From 12/2005 until 11/2017, 107 patients (55 female/52 male) were treated with combined RIT. Localization of lymphoma manifestations was supradiaphragmal in 41.1% of the patients (44/107) with mainly cervical involvement (72.7%) and infradiaphragmal in 58.9% (63/107) of the patients with mainly inguinal involvement (74.6%). Extranodal involvement was present in 20.6% of the patients, with most common manifestations in salivary glands (13/22 patients). In 13.1%, grade 3A FL were treated, which were excluded in the MIR study. Most of the initial lymphoma manifestations were small, with ≤7 cm (83%) and singular nodal/extranodal manifestations (56%); see Table 1. Ninety-five percent of the patients received all eight planned cycles of rituximab and RT was either applied with a 3D-conformal technique (71%) or intensity-modulated RT (IMRT; 29%). Representative RT plans are shown in Fig 1. Median time from first diagnosis to RIT was 4 months (0–25 months). Five patients with a longer period until the start of RIT (>12 months) were treated with a watch-and-wait strategy due to their own wish. Further patient and treatment characteristics are displayed in Table 1.

### Efficacy

**Fig. 2:**
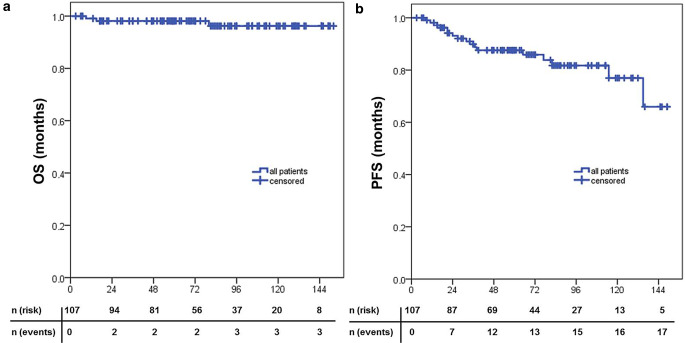
Kaplan–Meier survival curves after RIT.** a** overall survival (*OS*), **b** progression-free survival (*PFS*) in months for all lesions (*n* = 107)

Overall response rate (ORR) after four applications of rituximab was 78.8% (CR = 30.0%, PR = 48.8%). Seventeen patients (21.2%) were stable after rituximab application. At first FU 8–12 weeks after completion of RIT, ORR was 98.8% (CR = 71.3%, PR = 27.5%). ORR 6 months after RIT remained the same but with higher CR rates (91.3%). During treatment and until FU at 6 months, no recurrences were detected. Detailed and chronological information about response rates are displayed in Table 2 and Fig. 2.

**Table 2:** Chronological response rates after treatment

	Response after 4 × rituximab	Response at first follow-up after RIT	Response after 6 months
*Patients*	*n* = 80 (%)^a^	*n* = 80 (%)	*n* = 80 (%)
CR	24 (30.0)	57 (71.3)	73 (91.3)
PR	39 (48.8)	22 (27.5)	6 (7.5)
SD	17 (21.2)	1 (1.2)	1 (1.2)

*CR* complete remission, *PR* partial remission, *SD* stable disease, *RIT* radioimmunotherapy

^a^27 patients were not applicable, since lymphoma had been excised completely before start of treatment and were counted as CR

The reverse Kaplan–Meier estimate for median follow-up was 71 months (Q1–Q3 47–109; 95% CI 54.4–87.6) for PFS. Various patient, tumor, and treatment characteristics (age, sex, grading, stage, extranodal manifestation, localization, type of involvement, number of applied cycles of rituximab, RT technique) were analyzed as prognostic factors for PFS. None of those factors showed a significant correlation. Particularly nodal and extranodal manifestation (*p* = 0.428; HR 1.521; CI 0.54–4.33) and grade 1–2 vs. grade 3A FL (*p* = 0.159; HR 0.040; CI 0–43.98), characteristics that were excluded in the MIR trial, were not identified as negative factors.

Treatment response to rituximab (SD vs. CR/PR) before the start of radiotherapy (*p* = 0.015; HR 0.307; CI 0.11–0.83) was a parameter predictive for better PFS.

Tumor burden was a predictive factor as well, since patients with a manifestation <7 cm (*p* = 0.003; HR 0.260; CI 0.10–0.69) and patients with completely excised lymphomas showed better PFS (*p* = 0.038; HR 0.155; CI 0.02–0.89). Furthermore, multivariate analysis identified that tumor size <7 cm (*p* = 0.006; HR 0.223; CI 0.08–0.62) and response to rituximab (*p* = 0.041; HR 0.331; CI 0.12–0.91) were independent positive prognostic factors for PFS.

### Recurrence patterns and second-line treatments

During the whole FU, 17 patients (15.9%) suffered a relapse and were assessed individually. A detailed recurrence pattern analysis is shown in Table 3.

**Table 3:** Analysis of recurrence patterns after RIT for the 17 patients with recurrences

Patient (sex)	Age (years)	TTP (months)	Stage/grade	RT dose	Response to MIR Tx in week 7	Response after RIT	Recurrence	Size	Therapy	Outcome
1 (m)	65	36	II/1	40	PR	PR	Outfield	≤7 cm	L	PR
2 (f)	54	77	II/2	40	SD	CR	Outfield (DLBCL)	≤7 cm	R‑CHOP, HCT, SCTx	PR, †
3 (f)	52	24	II/1	40	SD	CR	Outfield	>7 cm	LDRT (2 × 2Gy)	CR
4 (f)	66	21	II/2	40	SD	PR	In-/outfield	>7 cm	IbTi	PR
5 (f)	47	27	I/2	30	n.a.	n.a.	Outfield	≤7 cm	W&W	CR (spontaneous)
6 (f)	58	37	II/2	30	CR	CR	Outfield	>7 cm	W&W	SD
7 (f)	65	8	I E (bone)/1	40	SD	SD	Outfield	>7 cm	RT (40 Gy) + R‑B	CR
8 (f)	63	17	II/1	40	SD	PR	Outfield	>7 cm	R‑B	PR
9 (f)	64	115	I E (bone)/1	40	SD	PR	Infield	>7 cm	R‑B	CR
10 (m)	82	82	I/2	40	PR	PR	Outfield	>7 cm	R	PR
11 (f)	42	11	I/1	40	PR	CR	Outfield	>7 cm	LDRT (2 × 2Gy)	CR
12 (m)	42	15	II/1	40	SD	CR	In-/outfield	>7 cm	IbTi	CR
13 (m)	54	38	I/2	40	PR	CR	Outfield	>7 cm	R‑B	PR
14 (m)	50	21	I/1	40	PR	PR	Outfield	≤7 cm	R‑B, HCT, SCTx	CR
15 (m)	65	135	I/1	40	PR	CR	In-/outfield	≤7 cm	R‑CHOP	CR
16 (f)	54	32	II E (bowl)/1	40	PR	CR	Outfield	≤7 cm	R‑B	CR
17 (m)	71	65	I E (sinus)/1	40	PR	PR	Outfield	≤7 cm	R	PR

*TTP* time to progression, *R‑CHOP* rituximab, cyclophosphamide, hydroxydaunorubicin, oncovin, prednisone, *E* extranodal, *HCT* high-dose chemotherapy, *SCTx* stem cell transplantation, *W&W* watch and wait, *R‑Benda* rituximab-benamustine, *L* lenalidomide, *IbTi* ibritumomab tiuxetan, *CR* complete remission, *PR* partial remission, *SD* stable disease, *PD* progressive disease, *n.a.* not applicable (no residual lymphoma before start of treatment), *DLBCL* large B‑cell lymphoma, *†* dead

Most of the relapses were outfield recurrences (94.1%). Three patients showed combined in- and outfield recurrences (17.6%) and only one patient developed an isolated infield recurrence (5.9%), leading to 2‑ and 5‑year PFS rates of 92.9% and 87.3%. In the whole cohort, only one patient (0.9%) suffered from histological transformation to diffuse large B‑cell lymphoma (DLBCL). Median time to progression (TTP) was 32 months (range 8–135 months). Regarding the patient cohort with recurrences, only two patients showed a CR after rituximab and before RT, so that 88% of the patients either had a PR or SD and were therefore treated with a higher radiation dose of 40 Gy.

Second-line treatment was applied in most of the cases (88.2%) with systemic chemotherapy/immunotherapy only (70.6%) or reirradiation (17.7%), either with a low-dose radiotherapy (LDRT) regimen of 2 × 2 Gy (11.8%) or 20 × 2 Gy (5.9%). Two patients were followed up regularly with a watch-and-wait strategy. After second-line treatment, all patients showed a response, with 53.3% CR and 46.7% PR to the time of this analysis (median of 67 months with range 3–133 months).

The reverse Kaplan–Meier estimate for median follow-up was 75 months (Q1–3 53–113; 95% CI 62.3–87.7) for OS. In total, three patients died during follow-up, leading to a 2- and 5‑year OS of 98.1%. Cause of death was not associated with the primary oncological disease or RIT treatment in any patient. One patient died due to skeptical organ failure after stem-cell transplantation due to an aggressive NHL (DLBCL, patient no. 2 in Table 3). Two patients died due to chronic heart failure.

### Toxicity

RIT was tolerated well, with mild (grade 1–2) acute side effects in 85.0% of the patients and only 0.9% grade 3 toxicity (one patient with acute mucositis, which needed temporary medical intervention). Most common acute side effects were dermatitis (34.8%) and mucositis (16.3%).

Most of the acute side effects resolved during further follow-up, so that 6 months after radiotherapy, 86.9% of the patients showed no further symptoms and only 13.1% of the patients complained about grade 1 late side effects, mainly xerostomia (40%).

Detailed toxicity criteria are shown in Table 4.

**Table 4:** Toxicity

Grade	Acute toxicity *n* (%)	Late toxicity *n* (%)
0	15 (14.0)	93 (86.9)
1	58 (54.2)	14 (13.1)
2	33 (30.8)	0
3	1 (0.9)	0
4	0	0
5	0	0

Toxicity according to CTCAE criteria

## Discussion

Treatment of early-stage FL has been discussed controversially over the past decades. Different treatment approaches are possible, depending on age and general condition of the patient. For elderly patients or patients in a reduced general condition, active surveillance might be considered.

Upfront radiotherapy has proven to be effective and improved both disease-specific and overall survival based on a large National Cancer Data Base analysis [20]. Several trials, among others the ARO 98-01 trial, used large treatment fields and showed a higher effectiveness compared to smaller irradiation fields, since most recurrences developed outside of irradiated areas [3, 7, 21], implicating that occult microscopic disease might be present in early-stage FL patients. Thus, several studies showed that combined treatment comprising radiotherapy and systemic chemotherapy and/or immunotherapy with rituximab were beneficial over radiotherapy alone, but with high toxicity rates [9, 13, 22, 23]. MacManus et al. reported on a high grade 3 toxicity rate after systemic therapy of 51% [13].

In the MIR study, the efficacy of combined IF-RT with rituximab was investigated and 5‑year PFS of the MacManus trial and the MIR trial were comparable at 86% and 78%, respectively, with considerably lower toxicity rates after RIT (51% vs. 4% grade 3 toxicity) [13, 15, 16]. Although the current analysis has its limitations owing to the retrospective nature, it confirms the results of the MIR trial with a comparable 5‑year PFS of 87.6%. Inclusion of extranodal disease or grade 3A FL were not identified as negative factors in covariate analysis and were not associated with inferior PFS. The MIR study excluded patients with FL grade 3A. The current data strengthen the hypothesis that patients with FL grade 3A might also benefit from the combined treatment approach as do patients with FL grade 1 or 2.

Furthermore, rituximab enhances radiation sensitivity in vitro [24] and several studies proved that rituximab eliminates minimal residual disease (MRD) [25, 26], underlining an abscopal effect. A limitation of this study is that MRD was not assessed and several studies recently emphasized the importance of molecular disease monitoring as positive bone marrow PCR at baseline highly correlates with poorer outcome [15, 26].

We could identify several positive prognostic factors for PFS in covariate analysis: response to rituximab after four cycles and after combined RIT were both associated with higher PFS rates. Response to rituximab might therefore be a useful parameter for a response-adapted treatment. Furthermore, patients with lower tumor burden (tumor size <7 cm, which was an inclusion criterion in the MIR study) and completely excised lymphoma manifestation showed better PFS. Considering this patient cohort, a further de-escalation in treatment regarding dose might also be an option, which should be investigated in the future. The fact that patients treated with 40 Gy suffered from progression more often is probably due to the fact that they showed an inferior response to upfront immunotherapy and that the higher tumor burden negatively affected outcome (which is also shown in the multivariate analysis).

Nevertheless, results of univariate and multivariate analysis have to be regarded with caution, due to the small number of events.

The recurrence pattern analysis shows that second-line treatment remains an effective option in recurrent disease, with a CR and PR rate of 53.3% and 46.7%, respectively. As already shown by our group, LDRT might be an effective and potentially curative option in recurrent disease, with long-lasting remissions [27].

Similar to the MIR study, most of the recurrences were detected outfield, emphasizing the importance of systemic therapy on the one hand but also the effectiveness of local radiotherapy on the other. Also, ORR after rituximab increased from 78.1 to 98.7% after RIT, showing the additional effect of RT.

The pivotal question of whether a lower radiation dose would also show the same efficacy is not yet resolved. Based on the publication of Lowry et al. [28], many consider a dose of 24 Gy to be sufficient. However, the latest prospective reports about the combination of local radiotherapy and systemic therapy used 30–40 Gy [13, 15].

Moreover, there have been several retrospective analyses that evaluated low-dose radiotherapy (LDRT) regimens with 2 × 2 Gy in a retrospective manner: all publications showed high ORR, but most of the patients were treated in mainly a palliative setting [29, 30]. Our research group just recently reported about the high efficacy, with a 93% ORR for primarily treated patients with indolent lymphomas after LDRT. In this cohort, 26% of the patients received rituximab simultaneously and showed no recurrence during the follow-up period [27]. A prospective German multicenter study (GAZAI study) that will investigate LDRT of 4 Gy in combination with the CD20 antibody Obinutuzumab in patients with early-stage nodal follicular lymphoma is currently ongoing [31]. This study will investigate a response-adapted treatment approach considering response to rituximab upfront, which this analysis has shown to be a prognostic factor for PFS.

For certain extranodal lymphomas, e.g., orbital lymphomas, LDRT only is associated with excellent control rates with very low local and distant recurrence rates [32, 33], so that additional rituximab in this patient cohort will probably yield only small beneficial effects.

Current guidelines recommend FDG-PET for staging purposes due to the higher sensitivity and specificity [34–36], because a remarkable number of patients show a stage shift when performing FDG-PET [37]. The FOLL05 trial identified an increased number of nodal involvement in 32% of FDG-PET staged patients and the impact of FDG-PET staging was highest with limited stages (62% upstaging with FDG-PET) [37].

Furthermore, two recent studies emphasized the prognostic role of FDG-PET-CT regarding outcome. On the one hand, outcome appears to be better for patients having received PET-CT compared to historical series (5-year PFS and OS 69% and 96%) [38]. On the other hand, Batlevi et al. evaluated 1088 patients and reported that the risk of histologic transformation was significantly higher in CT-staged compared to PET-CT-staged patients, with a 9.7-fold increase of death. Furthermore, PET-staged patients suffering from early progression had superior OS compared to patients who were staged with CT only (5-year OS 100% vs. 54%) [39]. The above-mentioned studies underline the strong recommendation and the prognostic impact of PET-CT staging in FL. Nevertheless, PET-CT staging was not used on a standard basis in our cohort. Therefore, patients with higher stages might have also been included and outcome data after combined RIT may even be superior. As a subsequent trial, the GAZAI study uses FDG-PET for staging as well as for (metabolic) response evaluation and therapy adaption.

The MIR study showed that combined RIT is tolerated well, with low toxicity and without compromising quality of life [15]. Although evaluated retrospectively, acute toxicity rates in our analysis were low, with mostly mild (grade 1–2) acute side effects in 84.5% of the patients and only 1.8% grade 3 toxicity (4% grade 3 toxicity in the MIR study).

A retrospective study compared involved regional RT to a smaller involved-site RT in 237 patients. The most common pattern of failure was distant failure, but with no difference after 10 years between the RT volumes (regional RT 38%, involved site 32%) and a 5-year PFS of about 65–70% [40]. Hence, reducing RT fields may not compromise long-term outcomes and the International Lymphoma Radiation Oncology Group (ILROG) also adopted this in their current guideline [41]. The superior 5‑year PFS rate in our analysis (87.3%) might be due to the advantageous treatment of combined RIT.

## Conclusion

Combined RIT in follicular lymphoma is an effective treatment option, with high response rates and long-lasting remissions as well as low toxicity rates. Although this is a retrospective analysis, we were able to confirm the results of the MIR study under real-world conditions and, furthermore, also investigated the efficacy of RIT in extranodal manifestations and WHO grade 3A FL.

## References

[1] Dreyling M, Ghielmini M, Rule S, Salles G, Vitolo U, Ladetto M, Committee EG (2017). Newly diagnosed and relapsed follicular lymphoma: ESMO clinical practice guidelines for diagnosis, treatment and follow-up. Ann Oncol.

[2] Lawrence TS, Urba WJ, Steinberg SM, Sundeen JT, Cossman J, Young RC, Glatstein E (1988). Retrospective analysis of stage I and II indolent lymphomas at the national cancer institute. Int J Radiat Oncol Biol Phys.

[3] Mac Manus MP, Hoppe RT (1996). Is radiotherapy curative for stage I and II low-grade follicular lymphoma? Results of a long-term follow-up study of patients treated at Stanford University. J Clin Oncol.

[4] Ott OJ, Rodel C, Gramatzki M, Niedobitek G, Sauer R, Grabenbauer GG (2003). Radiotherapy for stage I–III nodal low-grade non-Hodgkin’s lymphoma. Strahlenther Onkol.

[5] Neumann H, Blanck H, Koch R, Fiedler S, Lesche A, Herrmann T (2003). Follicle centre lymphoma: treatment results for stage I and II. Strahlenther Onkol.

[6] Soubeyran P, Eghbali H, Bonichon F, Coindre JM, Richaud P, Hoerni B (1988). Localized follicular lymphomas: prognosis and survival of stages I and II in a retrospective series of 103 patients. Radiother Oncol.

[7] Stuschke M, Hoederath A, Sack H, Potter R, Muller RP, Schulz U, Karstens J, Makoski HB (1997). Extended field and total central lymphatic radiotherapy in the treatment of early stage lymph node centroblastic-centrocytic lymphomas: results of a prospective multicenter study. Study Group NHL-frühe Stadien. Cancer.

[8] Wilder RB, Jones D, Tucker SL, Fuller LM, Ha CS, McLaughlin P, Hess MA, Cabanillas F, Cox JD (2001). Long-term results with radiotherapy for stage I–II follicular lymphomas. Int J Radiat Oncol Biol Phys.

[9] Kelsey SM, Newland AC, Hudson GV, Jelliffe AM (1994). A British national Lymphoma investigation randomised trial of single agent chlorambucil plus radiotherapy versus radiotherapy alone in low grade, localised non-Hodgkins lymphoma. Med Oncol.

[10] Landberg TG, Hakansson LG, Moller TR, Mattsson WK, Landys KE, Johansson BG, Killander DC, Molin BF, Westling PF, Lenner PH, Dahl OG (1979). CVP-remission-maintenance in stage I or II non-Hodgkin’s lymphomas: preliminary results of a randomized study. Cancer.

[11] Monfardini S, Banfi A, Bonadonna G, Rilke F, Milani F, Valagussa P, Lattuada A (1980). Improved five year survival after combined radiotherapy-chemotherapy for stage I–II non-Hodgkin’s lymphoma. Int J Radiat Oncol Biol Phys.

[12] Yahalom J, Varsos G, Fuks Z, Myers J, Clarkson BD, Straus DJ (1993). Adjuvant cyclophosphamide, doxorubicin, vincristine, and prednisone chemotherapy after radiation therapy in stage I low-grade and intermediate-grade non-Hodgkin lymphoma. Results of a prospective randomized study. Cancer.

[13] MacManus M, Fisher R, Roos D, O’Brien P, Macann A, Davis S, Tsang R, Christie D, McClure B, Joseph D, Jayamohan J, Seymour JF (2018). Randomized trial of systemic therapy after involved-field radiotherapy in patients with early-stage follicular lymphoma: TROG 99.03. J Clin Oncol.

[14] Skvortsova I, Skvortsov S, Popper BA, Haidenberger A, Saurer M, Gunkel AR, Zwierzina H, Lukas P (2006). Rituximab enhances radiation-triggered apoptosis in non-Hodgkin’s lymphoma cells via caspase-dependent and -independent mechanisms. J. Radiat. Res..

[15] Herfarth K, Borchmann P, Schnaidt S, Hohloch K, Budach V, Engelhard M, Viardot A, Engenhart-Cabillic R, Keller U, Reinartz G, Eich H-T, Witzens-Harig M, Hess CF, Dörken B, Dürig J, Wiegel T, Hiddemann W, Hoster E, Pott C, Dreyling M (2018). Rituximab with involved field irradiation for early-stage nodal follicular lymphoma: results of the MIR study. Hemasphere.

[16] Witzens-Harig M, Hensel M, Unterhalt M, Herfarth K (2011). Treatment of limited stage follicular lymphoma with rituximab immunotherapy and involved field radiotherapy in a prospective multicenter phase II trial-MIR trial. BMC Cancer.

[17] Cheson BD, Pfistner B, Juweid ME, Gascoyne RD, Specht L, Horning SJ, Coiffier B, Fisher RI, Hagenbeek A, Zucca E, Rosen ST, Stroobants S, Lister TA, Hoppe RT, Dreyling M, Tobinai K, Vose JM, Connors JM, Federico M, Diehl V, International Harmonization Project on Lymphoma (2007). Revised response criteria for malignant lymphoma. J Clin Oncol.

[18] Cheson BD, Horning SJ, Coiffier B, Shipp MA, Fisher RI, Connors JM, Lister TA, Vose J, Grillo-Lopez A, Hagenbeek A, Cabanillas F, Klippensten D, Hiddemann W, Castellino R, Harris NL, Armitage JO, Carter W, Hoppe R, Canellos GP (1999). Report of an international workshop to standardize response criteria for non-Hodgkin’s lymphomas. NCI sponsored international working group. J Clin Oncol.

[19] Schemper M, Smith TL (1996). A note on quantifying follow-up in studies of failure time. Control Clin Trials.

[20] Vargo JA, Gill BS, Balasubramani GK, Beriwal S (2015). What is the optimal management of early-stage low-grade follicular lymphoma in the modern era?. Cancer.

[21] Engelhard M, Unterhalt M, Hansmann M, Stuschke M (2011). Follicular lymphoma: curability by radiotherapy in limited stage nodal disease ? updated results of a randomized trial. Ann Oncol.

[22] Friedberg JW, Byrtek M, Link BK, Flowers C, Taylor M, Hainsworth J, Cerhan JR, Zelenetz AD, Hirata J, Miller TP (2012). Effectiveness of first-line management strategies for stage I follicular lymphoma: analysis of the national lymphocare study. J Clin Oncol.

[23] Seymour JF, Pro B, Fuller LM, Manning JT, Hagemeister FB, Romaguera J, Rodriguez MA, Ha CS, Smith TL, Ayala A, Hess M, Cox JD, Cabanillas F, McLaughlin P (2003). Long-term follow-up of a prospective study of combined modality therapy for stage I–II indolent non-Hodgkin’s lymphoma. J Clin Oncol.

[24] Skvortsova I, Popper BA, Skvortsov S, Saurer M, Auer T, Moser R, Kamleitner H, Zwierzina H, Lukas P (2005). Pretreatment with rituximab enhances radiosensitivity of non-Hodgkin’s lymphoma cells. J. Radiat. Res..

[25] Cencini E, Puccini B, Rigacci L, Fabbri A, Kovalchuk S, Mannelli L, Benelli G, Carfagno T, Simontacchi G, Bocchia M, Bosi A (2018). Radiotherapy plus rituximab as first-line regimen for localized follicular lymphoma. Leuk Lymphoma.

[26] Ruella M, Filippi AR, Bruna R, Di Russo A, Magni M, Caracciolo D, Passera R, Matteucci P, Di Nicola M, Corradini P, Parvis G, Gini G, Olivieri A, Ladetto M, Ricardi U, Tarella C, Devizzi L (2016). Addition of rituximab to involved-field radiation therapy prolongs progression-free survival in stage I–II follicular lymphoma: results of a multicenter study. Int J Radiat Oncol Biol Phys.

[27] Konig L, Horner-Rieber J, Bernhardt D, Hommertgen A, Rieken S, Debus J, Herfarth K (2018). Response rates and recurrence patterns after low-dose radiotherapy with 4 Gy in patients with low-grade lymphomas. Strahlenther Onkol.

[28] Lowry L, Smith P, Qian W, Falk S, Benstead K, Illidge T, Linch D, Robinson M, Jack A, Hoskin P (2011). Reduced dose radiotherapy for local control in non-Hodgkin lymphoma: a randomised phase III trial. Radiother Oncol.

[29] Girinsky T, Guillot-Vals D, Koscielny S, Cosset JM, Ganem G, Carde P, Monhonval M, Pereira R, Bosq J, Ribrag V, Vantelon JM, Munck JN (2001). A high and sustained response rate in refractory or relapsing low-grade lymphoma masses after low-dose radiation: analysis of predictive parameters of response to treatment. Int J Radiat Oncol Biol Phys.

[30] Haas RL, Poortmans P, de Jong D, Aleman BM, Dewit LG, Verheij M, Hart AA, van Oers MH, van der Hulst M, Baars JW, Bartelink H (2003). High response rates and lasting remissions after low-dose involved field radiotherapy in indolent lymphomas. J Clin Oncol.

[31] Konig L, Dreyling M, Durig J, Engelhard M, Hohloch K, Viardot A, Witzens-Harig M, Kieser M, Klapper W, Pott C, Herfarth K (2019). Therapy of nodal follicular lymphoma (WHO grade 1/2) in clinical stage I/II using response adapted involved site radiotherapy in combination with obinutuzumab (gazyvaro) – GAZAI trial (GAZyvaro and response adapted involved-site radiotherapy): a study protocol for a single-arm, non-randomized, open, national, multi-center phase II trial. Trials.

[32] Konig L, Stade R, Rieber J, Debus J, Herfarth K (2016). Radiotherapy of indolent orbital lymphomas : two radiation concepts. Strahlenther Onkol.

[33] Pinnix CC, Dabaja BS, Milgrom SA, Smith GL, Abou Z, Nastoupil L, Romaguera J, Turturro F, Fowler N, Fayad L, Westin J, Neelapu S, Fanale MA, Rodriguez MA, Hagemeister F, Lee HJ, Oki Y, Wang M, Samaniego F, Chi L, Esmaeli B (2017). Ultra-low-dose radiotherapy for definitive management of ocular adnexal B-cell lymphoma. Head Neck.

[34] Elstrom R, Guan L, Baker G, Nakhoda K, Vergilio JA, Zhuang H, Pitsilos S, Bagg A, Downs L, Mehrotra A, Kim S, Alavi A, Schuster SJ (2003). Utility of FDG-PET scanning in lymphoma by WHO classification. Blood.

[35] Tsukamoto N, Kojima M, Hasegawa M, Oriuchi N, Matsushima T, Yokohama A, Saitoh T, Handa H, Endo K, Murakami H (2007). The usefulness of (18)F-fluorodeoxyglucose positron emission tomography ((18)F-FDG-PET) and a comparison of (18)F-FDG-pet with (67)gallium scintigraphy in the evaluation of lymphoma: relation to histologic subtypes based on the World Health Organization classification. Cancer.

[36] Wohrer S, Jaeger U, Kletter K, Becherer A, Hauswirth A, Turetschek K, Raderer M, Hoffmann M (2006). 18F-fluoro-deoxy-glucose positron emission tomography (18F-FDG-PET) visualizes follicular lymphoma irrespective of grading. Ann Oncol.

[37] Luminari S, Biasoli I, Arcaini L, Versari A, Rusconi C, Merli F, Spina M, Ferreri AJ, Zinzani PL, Gallamini A, Mastronardi S, Boccomini C, Gaidano G, D’Arco AM, Di Raimondo F, Carella AM, Santoro A, Musto P, Federico M (2013). The use of FDG-PET in the initial staging of 142 patients with follicular lymphoma: a retrospective study from the FOLL05 randomized trial of the Fondazione Italiana Linfomi. Ann Oncol.

[38] Brady JL, Binkley MS, Hajj C, Chelius M, Chau K, Balogh A, Levis M, Filippi AR, Jones M, Mac Manus M, Wirth A, Oguchi M, Vistisen AK, Andraos TY, Ng AK, Aleman BMP, Choi SH, Kirova Y, Hardy S, Reinartz G, Eich HT, Bratman SV, Constine LS, Suh CO, Dabaja B, El-Galaly TC, Hodgson DC, Ricardi U, Yahalom J, Hoppe RT, Mikhaeel NG (2019). Definitive radiotherapy for localized follicular lymphoma staged by (18)F-FDG PET-CT: a collaborative study by ILROG. Blood.

[39] Batlevi CL, Sha F, Alperovich A, Ni A, Smith K, Ying Z, Gerecitano JF, Hamlin PA, Horwitz SM, Joffe E, Kumar A, Matasar MJ, Moskowitz AJ, Moskowitz CH, Noy A, Owens C, Palomba LM, Straus D, von Keudell G, Zelenetz AD, Seshan VE, Luminari S, Marcheselli L, Federico M, Younes A (2020). Positron-emission tomography-based staging reduces the prognostic impact of early disease progression in patients with follicular lymphoma. Eur J Cancer.

[40] Campbell BA, Voss N, Woods R, Gascoyne RD, Morris J, Pickles T, Connors JM, Savage KJ (2010). Long-term outcomes for patients with limited stage follicular lymphoma: involved regional radiotherapy versus involved node radiotherapy. Cancer.

[41] Illidge T, Specht L, Yahalom J, Aleman B, Berthelsen AK, Constine L, Dabaja B, Dharmarajan K, Ng A, Ricardi U, Wirth A, International Lymphoma Radiation Oncology Group (2014). Modern radiation therapy for nodal non-Hodgkin lymphoma-target definition and dose guidelines from the international lymphoma radiation oncology group. Int J Radiat Oncol Biol Phys.

